# Implications of Confidential Unit Exclusion in Providing Sufficient Safe Blood for the National Health System

**DOI:** 10.5812/kowsar.1735143X.765

**Published:** 2011-11-30

**Authors:** Abdol Majid Cheraghali

**Affiliations:** 1 Iran Blood Transfusion Organization Research Center, Tehran, Iran; 2 Department of Pharmacology, Baqiyatallah University of Medical Sciences, Tehran, Iran

**Keywords:** Iran, Blood Transfusion, Blood Safety

Blood transfusion at present and for the foreseeable future will be an essential component of health care systems. Blood transfusion is one of the most important lifesaving medical interventions which save millions of lives each year. Constant needs of patients waiting for surgery or suffering from trauma, severe anemia or complications of pregnancy to the blood and/or its components which necessitates the availability of timely and safe blood transfusion in healthcare facilities [1]. Despite the presence of substantial concerns regarding the safety of blood transfusion practices in some countries [2], recent achievements in the field of transfusion safety have provided fruitful results. The world's blood supply has never been as safe as it is today. During the past several decades dramatic progress has been made in reducing the risk of transfusion transmitted infections (TTI). In some countries this risk has been reduced to a negligible level [3]. These achievements can mainly be credited to improvements in policy and structure, recruitment of volunteer donors, the implementation of basic concepts of quality assurance in blood services and the use of high level technology in blood transfusion practices. In addition to these universal interventions some countries have also implemented interventions specific to their own national health systems.

Following US Food and Drug Administration (FDA) recommendations in the 1980s, some blood establishments inside the US and in a number of other countries have implemented the Confidential Unit Exclusion (CUE) method as a way to improve blood safety in their establishments. In this intervention donors may confidentially request that the blood service discards their donated blood after it has been donated, due to possible safety risks to the recipients from their blood donation. Although FDA recommendations for the implementation of CUE in the 1980s as a method to improve blood safety could be justified on the grounds of absence of reliable laboratory testing for HIV at that time, it withdrew its recommendation at a later stage. Therefore, due to the unproven efficacy of this intervention in improving blood safety, enthusiasm regarding the CUE method faded before its universal implementation and many authors seriously questioned its efficacy in improving blood safety. These reports have weighed the benefits of CUE against its main disadvantage, which is the discarding of a considerable number of donated blood units[4]. Investigators mentioned that despite the minimal effect of the CUE method in improving blood safety profiles, it contributes to a substantial amount of unnecessary wastage of donated blood units. This phenomenon is more pronounced in developing countries where low educated donors make up the largest proportion of the donors' pool. Ironically in these countries a shortage of blood is also more prevalent and providing sufficient blood is a challenging task. In some developed countries which have implemented the CUE option, the donor usage rate of CUE averages 0.5% or less, while this figure in developing countries is about 1%. This might indicate that the use of CUE in these countries is somehow tied to misunderstandings by donors regarding CUE's purpose and implications. A unit of blood is a scarce and precious lifesaving gift from donors and should be regarded accordingly by blood services. Therefore donated blood should only be discarded on the basis of a confirmed or at least justified presence of risk to the donors. Although there is a general consensus that volunteer donors will provide the safest blood donations, in countries which lack sufficient volunteer donors, recruitment of replacement donors is considered to be a practical approach for providing sufficient blood [5].

Iran is among the countries which has very reliable national blood transfusion services (2). CUE has been implemented in Iran's blood-transfusion system since 2003. However, recently the effectiveness of the CUE method in Iran's national blood transfusion service has been investigated. Results of these investigations and subsequent debates have been published in "Hepatitis Monthly" (4, 6, 7). In one of these papers the authors reported a significantly higher risk of HBV and HCV markers in donors who used the CUE option. Therefore, the authors concluded that CUE is an effective option for identifying donors with an increased risk of HBV and HCV infection (6). However, even in the data presented in this paper over 94% of HBsAg positive donations came from donors who did not use the CUE option to exclude their donation from being used. This paper has not reported the sensitivity and Positive Predictive Value (PPV) for the presented data. Therefore, based on this study alone it is very difficult to measure the impact of CUE on blood safety.

However, most of the other international studies have reported only low, albeit variable, sensitivity and PPV for the CUE method. Variability could be attributed to different methods of CUE application and the educational status of the blood donors. The CUE method is very much dependent on its application procedure. It has been reported that a small change in application methods of the CUE has drastically modified the frequency that CUE is used by blood donors. One study reported that modifying the procedure for donors' use of CUE and, more importantly, providing clear and self-explanatory CUE forms had a significant effect on the CUE usage rate. Such modifications have reduced the use of CUE in Germany by more than 30% [8]. In a recent published paper Farhadi et al. evaluated the efficacy of CUE in a Tehran blood center [7]. They concluded that the CUE option had low sensitivity and PPV. It had minimal effectiveness in reducing transmissible infectious diseases through window period units. In this study first time donors and donors who had a low level of education used the CUE option more often compared to repeat donors. This might indicate that these groups of donors are not sufficiently aware of CUE and its implications. However, they did not find a significant difference for positive test results for viral markers between CUE and non-CUE user donors. In this study more than 97% of donations with confirmed HBSAg positive test results were from non-CUE user donors. The fact that the majority of positive donated bloods for TTI came from non-CUE user donors emphasizes the importance of suitable donor screening methods instead of relying on the use of CUE by high-risk donors.

Although there is no robust evidence for improving the safety of blood transfusions as a result of CUE implementation in Iran, the universal application of CUE in all Iranian blood services has caused the unnecessary wastage of tens of thousands of donated blood units. In practical terms CUE should be designed carefully in order to exclude blood samples from high-risk donors who seek safety tests through transfusion services. These clearly indicate that the use of better donor screening and selection methods compared with the use of CUE, might be more efficient in excluding high risks donors from donating blood and prevent the unnecessary wastage of blood donations. Since the reported data shows that the CUE method is not as efficient as it was intended to be, especially in countries with a high rate of CUE use, such as Iran, it is important that both the procedures and donors' motivations for use of the CUE are investigated thoroughly.

In conclusion, although the CUE may modestly enhance blood safety in some blood transfusion services, this occurs at the cost of discarding a substantial number of blood units. Therefore, the CUE option should be re-evaluated in the broader national spectrum of blood transfusion services. Providing sufficient blood is of as much importance as the safety of the blood especially for those patients who are in urgent need of blood for life saving medical interventions. Patient's access to sufficient blood is a basic medical "right". Therefore policy makers should implement a balanced national blood policy that neither compromises the safety nor the availability of the blood and blood components
